# Caries Café: A Method for Consolidating and Co‐Constructing Knowledge of Caries

**DOI:** 10.1111/eje.70129

**Published:** 2026-03-09

**Authors:** Luisa Wakeling, Lucie England, Zoe Freeman, Helen Rogers, Greig Taylor

**Affiliations:** ^1^ School of Dental Sciences Newcastle University Newcastle upon Tyne UK; ^2^ School of Psychology Newcastle University Newcastle upon Tyne UK

**Keywords:** caries education, collaborative learning, educational intervention

## Abstract

**Introduction:**

Dental caries is multifaceted and requires dental undergraduates to receive training in underlying science, risk factors, disease prevention, and treatment. Caries content features at many different points in a Bachelor of Dental Surgery (BDS) programme, making it a large task to consolidate and assimilate in clinical practice. The study aim is to investigate how effective a collaborative educational intervention is in consolidating taught content on dental caries and integrating related clinical experience through co‐construction.

**Methods:**

A collaborative idea generation tool was adapted using social constructivist principles as an educational intervention to consolidate and co‐construct pre‐clinical and early clinical caries content. Students visit a series of tables, answering questions through divergent thinking around aspects of caries. A qualitative study aligned to utilisation focused evaluation was conducted through four focus groups: two with students who recently received the intervention, and two with final‐year students. Transcripts were analysed using reflexive thematic analysis.

**Results:**

Thematic analysis generated themes: Consolidation and co‐construction of knowledge of caries; Learning opportunities within Caries Café, Delivery of Caries Café, and Improvements to Caries Café. The café promoted collective working, while permitting individuals to ask questions. Overall confidence in caries identification increased, but further clinical reference is preferred by students for potential longer‐term benefits.

**Conclusion:**

The Café format served as a method to consolidate and co‐construct caries knowledge in a programme where content is dispersed and experiences varied. It provided holistic exposure to a multi‐faceted subject, with question topics aligned to domains of a proposed standardised caries curriculum.

## 1 Introduction

Dental caries is the most prevalent chronic oral condition observed globally [1]. Despite this, there is currently no standard approach to teaching caries internationally, both theoretically and clinically. A standardised, evidence‐based core curriculum in cariology (CCC) was developed in 2010 by the Organisation for Caries Research (ORCA) [2]. However, there are still challenges to the widespread implementation of this framework. The CCC sets out the learning outcomes for undergraduate training, but it does not determine how this curriculum should be implemented, leaving it open to individual institutions to determine how best to deliver this. Outdated techniques continue to remain in undergraduate education, with advancements often limited to theoretical teaching [2, 3, 4], which results in a lack of educational alignment to clinical guidelines [5] and students underprepared in managing conditions related to caries [6]. Furthermore, there are noted gaps in the evidence base, such as the management of early, cavitated carious lesions, which was highlighted by a recent scoping review of caries guidelines [5].

Cariology is not always considered a recognised discipline, and this broad, multifaceted topic is dispersed across theoretical, preclinical, and clinical delivery within a Bachelor of Dental Surgery (BDS) programme [7]. Content covered includes underlying science, risk factors, aetiologies, and prevention and treatment, nuanced by variations across different patient groups. Limited integration of cariology science and clinical dentistry is a recognised problem, with limited educator training and support for non‐operative caries management, thus delaying an important shift from disease focus to that of prevention [2, 4]. In pre‐clinical years, caries content is often delivered in a traditional lecture‐based programme. Students' clinical experiences of managing caries vary. The many aspects of caries teaching can be isolated in the learning process, and consolidation of the learning is often not undertaken. In our context, there is no package of learning dedicated to caries, a feature that is also common across European dental schools [7].

With the disconnected nature of learning about caries, the lack of an internal caries curriculum (e.g., module, course or strand) and that each learner can construct knowledge differently from various contexts, a need was identified by the research team to consolidate and integrate knowledge of caries in the curriculum [6]. There is an increase in collaborative case‐based discussion and active learning in clinical education with a view to ensure health care professionals can consolidate and integrate knowledge of the wider subject area [8]. This collaborative approach can also facilitate students to construct new knowledge and insights together in a socio‐constructivist model of learning [8, 9]. An educational intervention based on a social constructivist learning environment was designed and led following the principles from Adams [10]: a focus on learning not performance; learners as co‐constructors of meaning and knowledge; teachers as learning guides not instructors; and learners engaged in tasks seen as ends in themselves and consequently, as having implicit worth. The collaborative idea generation tool, World Café [11], has been used in healthcare by professionals and key stakeholders for qualitative data gathering [12] and by educators to facilitate communities of practice to discuss large, overarching topics in health education [13]. Used previously in dental education as a social and academic induction event for 1st year students at Newcastle University [14], the café method lends itself to implementing principles of a social constructivist learning environment and was adapted to consolidate caries learning across the first 3 years in the 5‐year BDS programme. Caries café has been delivered to students in year 3 of the BDS programme at Newcastle University since 2016. The need to formally evaluate this educational intervention was evident to determine if the design of the café fulfilled its purpose. The aim of this qualitative study is to investigate the following research question: does a collaborative educational intervention support consolidation of taught content on dental caries and integration of related clinical experience, through co‐construction?

## 2 Methods

Newcastle University's Student Employment on Campus scheme supported the research position held by Psychology student L.E. Z.F., G.T., H.R. and L.W. are a diverse research team in the School of Dental Sciences who all teach in the Caries Café session and across the BDS programme; Z.F., G.T. and H.R. are clinical paediatric dentists focusing on different areas of research related to dental education, health economics and health inequity, and cariology and the curriculum respectively. L.W. is a preclinical science senior lecturer focusing on dental education and health inequity. Ethical application for the study was accepted by Newcastle University Research Ethics Committee (Ref: 24172/2022) and work followed the British Educational Research Association guidelines [15]. The Caries Café educational intervention will be described followed by methods for evaluation. Figure 1 shows a flowchart of the method followed.

**FIGURE 1:**
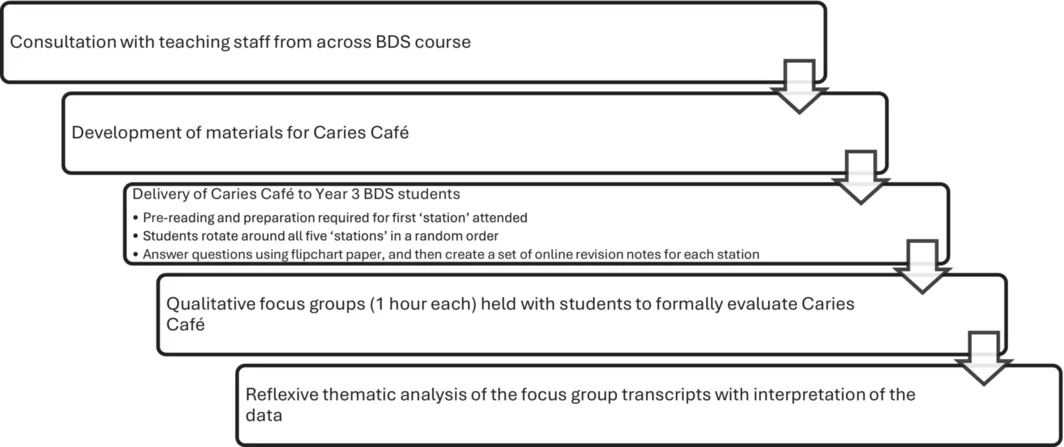
A flow chart detailing the methods followed in this study.

### 2.1 Educational Intervention

Year 3 students were chosen to undertake Caries Café in term two, permitting integration of preclinical teaching and early clinical experiences. Students visit five tables (stations) answering questions that promote divergent thinking (Table 1), focusing on different aspects of caries that encourage recognition of long‐term value such as exploring novel future caries management strategies and developing a bid for funds for an oral health initiative. Teachers from across the BDS programme were consulted for the questions that would cover most aspects of caries teaching and encourage discussion and integration of material. Each table is facilitated by a programme teacher who was briefed to observe, listen, be nonjudgemental and encourage student–student dialogue. Students rotate, in a random order, with approximately 6–8 students visiting each table per rotation. To set expectations of the session and encourage confidence in ability to address their first table's question only, students prepare by reading and sourcing relevant literature in advance of the session. Answers are built upon on flip chart paper by subsequent groups new to the table. The last group create a set of revision notes, using Chromebook technology, encapsulating knowledge and experiences of the entire year. Revision notes were validated by facilitators prior to dissemination.

**TABLE 1:** Example questions and activities from Caries Café with accompanying literature preparation required by students. Questions were aligned to the domains of a European Core Curriculum in Cariology (CCC) [16].

Station	Question	Intended learning, preparation, and associated literature	European Core Curriculum in Cariology (CCC) domains [17]
A	Looking back over the BDS programme content, can you identify factors and risks associated with the initiation and progression of caries?	The aim of this question was to support understanding of the multifactorial aetiology of caries; identification of risk factors in different patient populations; and encourage reflection on learning about the pathophysiological caries process whilst integrating disease prevention knowledge. The initial student group was to provide two pieces of resource for the table, e.g., slides, journal articles, information from the NHS and to read the article provided by staff [18]	Align to domains: I. The knowledge base; II. Risk assessment, diagnosis and synthesis
B	Develop a strategy for the translation of 3 new ideas for caries prevention into clinical or personal use	Students were encouraged to consider and develop strategies for different types of new approaches to preventative measures in dental care, e.g., chemical vs. physical vs. biological, individual‐level vs. population, children vs. adults. The initial student group were to provide two pieces of contemporary literature and to read the article provided by staff [19]	Align to domains: I. The knowledge base; III. Decision‐making and preventative non‐surgical therapy; V. Evidence‐based cariology in clinical and public health
C	Design a public health campaign to address childhood caries in the North East of England	The aim of this set of questions was to support understanding of health promotion in dental care and develop effective engagement and communication strategies in prevention. Each student group was to sequentially provide information that will help towards this campaign. The initial group started with the question, ‘As a group think about the impact that dental caries in children has upon individuals and local populations.’ Students were directed to read journal articles [17, 20]	Align to domains: I. The knowledge base; III. Decision‐making and preventative non‐surgical therapy; V. Evidence‐based cariology in clinical and public health
D	A patient has evidence of non‐cavitated white spot lesions. What could you do and/or recommend to encourage remineralisation? Look at the images of caries provided and discuss	Students were provided with images of caries and radiographs for 5 different patient cases at this table. This was to support the recognition of non‐cavitated white spot lesions and to recommend appropriate remineralisation techniques considering compliance at this early stage. The initial student group were to bring two pieces of resource for the table, e.g., slides, journal articles, information from the NHS and to read the article provided by staff [21]	Align to domains: I. The knowledge base; II. Risk assessment, diagnosis and synthesis; III. Decision‐making and preventative non‐surgical therapy; IV. Decision making and surgical therapy
E	Reflect on your caries learning experience and discuss areas of difficulty or gaps in knowledge or skills that could be brought to the curriculum	Students were to provide feedback on caries teaching in the BDS programme so far. This question aimed to identify areas of difficult or gaps in understanding related to caries management and encourage reflective practice. The initial group were to prepare by reviewing caries teaching across the programme and recalling their experiences with caries.	Align to domains: I. The knowledge base; II. Risk assessment, diagnosis and synthesis; III. Decision‐making and preventative non‐surgical therapy; IV. Decision making and surgical therapy; V. Evidence‐based cariology in clinical and public health

### 2.2 Evaluation

Caries Café has now run for several years and has been informally evaluated with students by in‐session feedback. Improvements have been iterative based on this feedback, such as the introduction of more clinical images of caries, reviewing questions that cause repetition, reducing stations, smaller groupings, and increased time at tables, though the request for coffee and cake at the Café has not yet been fulfilled. It was not known though if the educational design of the café was meeting its purpose. A formal evaluation, aligned to utilisation focused evaluation guidance [22] was carried out. Utilisation focused evaluation is a framework to support the effective evaluation of educational interventions in the health professions aligned to stakeholders' needs. This evaluation did not set out to measure the direct knowledge acquisition or clinical performance around caries, instead if the intervention is a solution to the educational problem of fragmented teaching and varied clinical experiences of caries and to utilise these findings to inform future iterations of the intervention.

This study employed a qualitative research study design using focus groups to explore the following evaluation questions:

- Does the design and delivery of the Caries Café facilitate the consolidation and integration of knowledge of caries aligned to the CCC?
- Does the design and delivery of the Caries Café support opportunities for co‐construction of new information?
- What is the student perception of the influence of the Café on learning about caries and are there any long‐term benefits?

Non‐dental student intern, L.E., conducted four student focus groups with the support of G.T. (a researcher with training and experience in qualitative methodologies) to evaluate the Caries Café. L.E. brought a student perspective to the research and enhanced engagement in focus groups with peers. Focus groups were conducted in familiar seminar rooms in the dental school or online. All year 3 (1 month post intervention) and year 5 students (1 years post intervention) were invited by email through the general office and given participant information sheets. Convenience sampling was used. Written consent was obtained. Each received a £10 voucher for participation.

The topic guide was co‐developed by all authors utilising the informal student feedback from seven previous café sessions thereby including a dental student stakeholder perspective in the evaluation. Broad exploratory areas of the guide were: memories of the café, feedback of the session and knowledge of caries. Focus groups of approximately 1 h each were audio recorded and transcribed verbatim by L.E. Transcripts were re‐listened to ensure accuracy of transcription. The methodological approach of reflexive thematic analysis [23] was chosen to analyse the transcripts where data could be interrogated for patterns and meaning across many student responses and themes generated considering researcher interpretation. Reflexive thematic analysis within Microsoft Word began with all authors familiarising themselves with the data. L.E. was the primary coder and conducted inductive coding without preexisting notions about the café format or dental education, providing only a general student perspective. The research team (Z.F., G.T. and L.W.) then each looked at the transcripts with the proposed codes and noted codes and areas of the transcript for discussion. Through collaborative coding review meetings with L.E., the team ensured that the interpretations were reasonable, refined where necessary, resolved any disagreements and identified overlooked data. Codes were grouped into potential themes considering our reflexivity in the context and conduct of this research. The interpretation of the data therefore was influenced by the research teams' experience of the Café format, awareness of the BDS programme and other dental schools' curricula, clinical and non‐clinical experience and teaching undergraduate dental students. Themes were developed inductively from participants' accounts and not derived deductively from the evaluation questions. Questions that formed the stations of the café were also reviewed for aspects that relate to the recommended ‘domains’ of the CCC [2, 16].

## 3 Results

Three in‐person and one online (due to availability) focus groups were conducted. A total of nine year‐5 students and ten year‐3 students participated across four focus groups (*n* = 4–6). There was approximately a 50:50 male–female split across groups.

Four themes were generated from the data about the café: Consolidation and co‐construction of caries Knowledge; Learning opportunities within Caries Café; Delivery of Caries Café; and Improvements to Caries Café. Further subthemes and representative quotes supporting the themes can be found in Supplementary Tables 1–, 4.

### 3.1 Consolidation and Co‐Construction of Caries Knowledge

#### 3.1.1 Knowledge Integration and Consolidation

Across both year groups it was noted that overall, the café consolidated existing caries knowledge that was otherwise not evidently linked and supported translation of theoretical knowledge to practical application. This was deemed useful in clinics and perceived to increase confidence. This holistic approach facilitated integration and revision of the material and helped identify gaps in knowledge. The café was a good ‘stepping stone’ into clinics (Supplementary Table 1).

It was good to sort of get all the information in one place…generally we had the knowledge anyway, so it did feel like more of a revision session where you like bring everything together. I think a lot of times where you're going over topics, you'll learn like this here and this here, and you kind of separate them in your mind, but I think to bring them all into one place and look at it all in one time helps you to sort of, I guess, make all the connections between the two, between all the different areas (Year 3, Group 2, P6).

And it was just it was just a nice building block … especially in third year, I was very much like, baptism of fire on clinic. I didn't really know what was going on, I was like learning stuff and lectures at the same time trying to piece together, I was very reliant on the clinicians, and which is expected in only third year, but I think, at least with caries café at least helped understand, just like took bread and butter of what we're supposed to be doing. So again, a nice foundation, which I think only clinic can sort of build upon (Year 5, Group 4, P4).

#### 3.1.2 Knowledge Co‐Construction

This subtheme was not originally proposed by the student intern but instead included in the later subtheme of group work. The research team, reflecting on the purpose of the café design, felt it was an important theme around knowledge in the café. The fact that students were starting from different knowledge bases was acknowledged by them. There was a good amount of evidence to support the co‐construction of learning amongst students during the café which featured understanding caries from different perspectives, learning about new ideas, discussing different clinical options and opinions, and “filling in” knowledge gaps.

From third year till now we've only really been with our clinical groups, so you hear a lot about what people in your clinical group are doing on clinic I think you have different knowledge based on which clinical group we're in (Year 5, Group 4, P3).

…you get to talk to more people in the year group and hear loads of other people's perspectives on things rather than just all the same set of brains for all the different stuff…One person might have an idea that no one else has, if you stayed in one group you wouldn't have heard about that idea and it's better to [be] able to spread that information wide (Year 3, Group 2, P5).

…you know when you ask a question, then someone asks a question on top of that question, then when you think about that question you get an answer. It's more than you asked for but its better overall for you because you've gained more knowledge than you thought (Year 3, Group 3, P3).

### 3.2 Learning Opportunities Within Caries Café

#### 3.2.1 Beyond the Curriculum and Wider Implications of Caries

Year 3 students felt that Caries Café provided opportunities for new learning, as some stations prompted understanding from new perspectives and new technologies. Opportunities were also provided to learn more about the wider implications of this oral disease that had not previously been considered. There were opportunities for learning that impacted on the future: from advancements in research and public health to inclusion of clinically‐related elements that were noted as more useful for future practice, of which some year 5 students favoured over more academic knowledge base content (Supplementary Table 2).

I found one of the stations was about new things in dentistry and that was really interesting because we haven't really been taught about that …just to learn about new technologies that I didn't even know existed (Year 3, Group 2, P2).

The one about where it's like government health scheme one, I think that was quite helpful in some ways thinking about, the way, thinking outside of just a clinical setting, like what you think about regarding dentistry and things. I think it was interesting to think about it from that perspective (Year 3, Group 2, P4).

#### 3.2.2 Identifying Caries

It was evident that the use of clinical images on clinical stations was memorable in the long term, and in the shorter term, year 3 students felt visual aids improved their learning opportunity in the identification of caries for which they had little experience. For students within both cohorts, including clinical cases and images helped bridge the knowledge‐practical translational gap, and appeared to enhance the learning opportunity (Supplementary Table 2).

A lot of the time before I would be like ‘oh, it's either caries or a stain’, and I wasn't sure which one it was. Now I feel more comfortable being like, ‘that's just staining, that's caries’ (Year 3, Group 2, P6).

Related to this clinical aspect was also the importance of co‐construction

…someone was saying that they had attrition and could recognise that in the child's dental health image. It's like they said that now I know when I see something like that, it's cause they had it (Year 3, Group 2, P4).

### 3.3 Delivery of Caries Café

#### 3.3.1 Group Work

Positive components of the café design and delivery were recalled by several students stating that the session was fun and enjoyable and a good variety on the tables. There were also those that found it long and, though interesting, found some of the tables difficult to work through (Supplementary Table 3). Group work allowed students to exchange ideas and discuss complex topics. Students felt more comfortable conversing in smaller groups than they usually would in a lecture theatre, and the constant changing of tables and personnel each time permitted interactions with peers whom they would not routinely speak to. Group work also had its downsides, with uncertainty if all had completed the preparative work and sometimes affording an opportunity for some to sit back and let others answer. Some found doing the preparative work for their first question helpful; some admitted not doing it.

I feel like when there's six people in a group, I'm so happy to just answer (Year 3, Group 3, P2).

I don't know if there's any way of sort of moderating how the input everyone puts in beforehand. Because we had to do some prep I think as a group but there's always a group where some people don't do anything (Year 5 Group 1 P5).

#### 3.3.2 Learning Dynamics

The café delivery had an interesting influence on learning dynamics providing an informal environment to interact with expert staff members, something students felt less comfortable to do during a conventional lecture. The café environment therefore enables students to ask the ‘silly’ or smaller questions, facilitating learning. The ability to hear what others want to ask aids further consolidation and construction of information. Students appreciated meeting staff they hadn't yet encountered in the programme.

It was quite informal, and you could just ask what you wanted. Whereas in a lecture, you feel like you need to, kind of, phrase stuff more professionally (Year 3, Group 3, P3).

#### 3.3.3 Role of the Staff Facilitator

This was a subtheme for which the importance was recognised by the staff in the research team given their role in the café, but this was not originally put forward by the student intern. The role of the staff facilitator was key to keeping the discussion on track and provided the ability to ask questions easily, as well as allowing for lack of knowledge to be addressed in a timely, nonjudgemental manner. The close interaction with experts was valued by students and that staff could take on feedback about caries education was important.

I think the lecturers being there was helpful. I think it would have been very different if it was just student like chatting away yeah. It's good to like, have them there to know that you're on the right track and tell you the right answers if you get it wrong (Year 3, Group 3 P1).

Cause they've treated the patient as well, you can like ask them more questions (Year 3, Group 3, P4).

One of the stations…where they said ‘what do you think could be done to kind of improve your knowledge of caries and what have you sort of been taught so far on the course etcetera’. I think that's kind of really nice because it feels like they're taking on feedback from us and I think with that, quite a lot of people had quite a lot to say about it even like people that often wouldn't say something (Year 3, Group 3, P1).

### 3.4 Improvements to Caries Café

More of a minor area in the data but nonetheless important in evaluation, improvements to the café were identified. Despite being validated by a member of staff before dissemination, students preferred model answers over group‐generated revision notes. Students may be unaware of the staff validation process. Alternatively, they may mistrust the quality of the revision notes due to contributions by other students. This is an interesting dynamic given they acknowledged the value of peer discussion in the café. Incorporating additional clinically relevant stations, or clinical quizzes, into the café structure was desired. In discussion of changes to future cafés, the café format was deemed flexible for adaptation to other subject areas such as human diseases and treatment planning (Supplementary Table 4).

…what would kind of help me would be more clinical stuff. Because then that's not only just going to help me with exams but actually when we're there [in clinic] with confidence (Year 3, Group 3, P3).

I think it could be useful for anything. I think it really helps you draw any knowledge you have, drag it from the back of your mind when you're put in a situation where you have to talk about it. I think it's useful for so much (Year 3, Group 3, P1).

## 4 Discussion

To our knowledge this is the only café‐style method utilised for caries teaching differing from the conventional and traditional methods. While similar to the principles of a flipped learning approach, including social constructivism as an underpinning theory [24], the café methodology uses only partial preparation directly before the session and draws on extensive prior learning over a longer period compared to typical flipped classrooms. It is designed to be delivered at a key stage in the transition from preclinical to clinical learning and not as a replacement for regular didactic delivery. It is also on a larger scale in terms of in‐session subject level coverage and facilitator input.

This qualitative formal evaluation of Caries Café determined the café design served as a strong method to enhance consolidation and integration of caries knowledge. Consolidation and integration of topics are fundamental to learning as denoted by Bruner's [25] spiral curriculum, helping students deepen their knowledge, reinforce long term memory of topics and support application. Students clearly recognised that the café provides a space to revisit information about caries; build on prior knowledge and allow for the expansion of that knowledge; and a ‘stepping stone’ to clinical activity. Re‐encountering cariology content with clinical relevance, particularly at this transition point, positions the café as an integrative learning activity that may bridge the gap between theoretical understanding and has the potential to improve diagnosis and clinical decision making [26]. Integration in dental curricula can be challenging and so this may be a way to support covering a broad topic area across subject disciplines [27]. Students felt this method could be adapted to other diseases such as periodontal disease or other broad topic areas such as dental trauma and treatment planning and so this may raise awareness of more spiral‐based strategies to learning across the programme.

The café achieved holistic exposure to a multi‐faceted topic where all questions aligned to the domains of a proposed standardised caries curriculum [16] (Table 1). As the café questions were generated in consultation with staff who teach caries‐related content across the BDS programme, this provides insight into the internal curriculum suggesting that we are at least addressing the broad categories of cariology, but that the café is required to highlight, link and consolidate the content. Therefore, the café could support the organisation of knowledge for students as a kind of curriculum map for caries with respect to revealing to them links across subject areas that are not always visible via traditional teaching approaches. Supported by Bruner [25], linking topics is a powerful part of learning and facilitates thinking about the bigger picture.

Students also recognised positively the contribution of others in their discovery about caries. From the evaluation, the format of the café was found to be fulfilling its purpose with incidences of facilitating knowledge and understanding in a socially constructed way. The café permitted students as individuals to ask questions but also promoted collective working. Social interaction with staff and, importantly, peers is an integral part of constructing knowledge in learning, developing a deeper understanding and allowing the zone of proximal development to grow [28]. Students clearly valued the opportunity the café provided to explore the opinions of others, particularly from those in different clinical groups, whilst promoting divergent thinking with peers and facilitating staff and the ‘checking’ of their understanding by facilitators that they articulate may not be afforded in didactic teaching methods like lectures. The complementary nature of the café addresses the limitation of didactic teaching and allows for exposure to peers' early clinical experiences and prompts students to discuss clinical implications. Experiences like these that increase dialogue and comparing perspectives, may support students to begin developing a professional approach to communication and reasoning skills [29].

While the above findings demonstrate the value of the café far beyond that of a revision exercise, a main goal of the cohort was to generate a set of revision notes from the session. Revision materials generated by the students in the café appear useful in the short‐term but, despite the valued collaboration in the session, it was noted that including staff‐reviewed ‘ideal’ answers and case studies were preferable outputs that may increase usage long‐term. The advertisement that staff review and validate the notes generated has since been enhanced to address the concern over peer‐generated notes. The request from one student for introducing clinical quizzes into the café structure will be approached with caution as it conflicts with the focus on learning not performance [10].

In the collaborative learning environment, the quality of the interaction can be dependent on factors such as student training, group characteristics, the goal or incentive of the structured task, and the culture of dialogue [30]. While some of these areas have been supported with an early experience of a similar format [14], providing student preparation material, reducing group size and introducing more clinical relevance, there is still room for fine tuning of the system. Supporting the culture of dialogue between students has not yet been explored and is possibly a fundamental feature to the success of this collaborative working [30], with aspects such as reciprocal sense‐making and listening to others' lines of argumentation, important skills to bring. There was no direct evidence that raised issues with these particular aspects and in the main, there was a positive stance from students on the input of different opinions and ideas but students did pitch the downside of group work where there were dominant voices and those who perhaps did not do the preliminary work to enable them to contribute, which could be interpreted to impact negatively the culture of dialogue.

This leads to acknowledging the limitations of co‐constructivism and therefore the intervention. Though a desire for knowledge expansion, a shared understanding will never be entirely consistent, and students are exposed to different ideas and opinions within and across groups leading to further divergent thinking and outcomes [30, 31], It is felt that the role of the facilitator in correcting misunderstanding, building on previous group material and the convergence of the revision notes may have lessened inconsistency. Related to this, many participants in speaking of co‐construction revealed there being large quantities of different opinions and ideas. As noted above this was pitched in a positive way and is important to social construction of knowledge; however, this may have been a reason for some of the student difficulty where information overload was reported. For future pedagogical facilitation, working on the culture of dialogue may decelerate and enrich discussion of opinions and ideas, allowing for added prioritisation and convergence.

While we did not set out to measure direct learning of content from the café, it was indicated by students that it influenced their learning by:

- Facilitating learning opportunities for revision.
- Identification of knowledge gaps and introducing new technologies.
- Improving confidence in identifying and diagnosing caries.
- Encouraging thinking about the wider implications of caries in public health.

This influence on learning may be important in improving the knowledge acquisition and clinical management of caries by students, but this would require a different evaluation of the café.

In recent delivery of the café, specific prompts related to clinical management were introduced by a facilitator to one station. This addition was prompted by informal feedback indicating a learning gap and limited prior experience, which was also later confirmed by the current findings. Year 3 students noted they felt more confident in being able to identify caries after the café. This is an important aspect that the café highlights and is worth exploring further as it has been shown that students' decisions regarding caries treatment vary significantly depending on their exposure to diagnostic criteria [32]. Clinical experiences have also been shown to heavily influence decision making in caries management over time and so discussing clinical aspects with peers and clinical facilitators, as evidenced in our data, may improve understanding and consistency in decision making where variability can be adverse in clinical care [33].

Student feedback suggested that strengthening the case‐based content and caries recognition content of the café may facilitate sharing of early clinical experiences, allowing students to reflect on different approaches to caries management and apply this to their clinical practice up to year 5 and beyond. Despite differences in the amount of clinical experience, both cohorts from each year group suggested that the café content would be more beneficial if it related more to clinical practice. In dental education where there is known difficulty in updating curricula to reflect evidence‐based practices [4], the café space permitted exposure to real‐world topics that wouldn't be conventionally covered in a lecture, developing adaptability, a useful skill for future clinical practice. Solving problems that are like scenarios in their future career is an important later stage in the construction of knowledge for students where meaningful learning is encouraged by undertaking authentic tasks [31]. Further stations require review to maximise clinical content, potentially by centring around case‐studies.

A strength of the café, as appreciated by students, is the inclusion of teaching staff from different fields: clinical (from across different specialties) and non‐clinical (experts in a variety of pre‐clinical subjects), all facilitating discussions related to their area of expertise within the field of cariology. The teacher's broad expertise and experience were valued by students as well as the nonjudgemental and informal setting they created, factors that are promotive of a collaborative learning environment [10, 28, 30]. The questions supplied by teaching staff used in the café were also reviewed for their alignment to the five recommended ‘domains’ of the CCC [2, 25], representing the broad categories of cariology (Table 1). All questions aligned to at least two domains with complete coverage of all domains noted. Two of the major challenges to implementing a core curriculum in cariology are the separation between cariology and clinical dentistry and the focus on restorative/operative management, with prevention and nonoperative management being disconnected [2]. The café over the years has included facilitators utilising questions that have encouraged the discussion of biological management and innovative preventative qualities of materials, areas found in guidelines but not always in undergraduate teaching [5]. It would be of interest to explore with teaching staff if there were any changes to their approaches to caries teaching because of their involvement in the café, which can impact on clinical practice, where instructors are one of the most important factors in shaping the decision‐making of students in caries management [33].

This utilisation focused evaluation has provided the research team with key lessons and development opportunities for the café and its stakeholders. While the café does what it was intended to in providing the space and setup for consolidation and co construction, a focus on supporting the dialogue around learning is needed, which may increase collaboration within group work and reduce information overload. Facilitator and student training will be required to achieve this. Another aspect is to scaffold more of the session with increased clinical content for relevance and longer‐term impact. It is possible that more stations could be centred around clinical cases.

### 4.1 Limitations of the Study

The implementation and evaluation of this teaching method is set in one BDS programme, in one context. Interest in adoption of this method has been expressed by other dental schools and subsequent evaluation in an additional context has the potential to strengthen the findings. It was not the same students from year 3 to year 5 in the focus groups; it may have been more powerful in understanding the longer term effects and if their thoughts and utilisation of the café persisted or changed in a longitudinal approach. It is also important to acknowledge that the students who came forward for the focus groups were self‐selecting and may have been biased towards enjoying the session, though this did not prevent students admitting it was challenging. It would have been helpful to explore this further revealing if this difficulty is influenced by any learning differences, culture and the feeling of safety, reasons why students may be challenged in active learning sessions [34], though this was not appropriate in the focus group setting.

## 5 Conclusion

While Caries Café cannot fully compensate for a standardised core curriculum, it does link the domains of the CCC and fulfils the need for facilitating consolidation and co‐construction of learning about caries in a programme where content is dispersed and experiences varied. Caries management is so much more than operating on the cavity, and providing space in the curriculum to discuss variability and advanced ways of caries management is an important aspect to this educational intervention. This evaluation has determined that Caries Café is valuable to students' learning opportunities and will continue to occur in the BDS programme. This work has the potential to inform others who wish to address the consolidation and integration of multifaceted topics for which there is no dedicated package of learning within a programme.

## Author Contributions

Lucie England contributed to the data collection and analysis, manuscript preparation, and was an undergraduate student intern at the time of the work. Zoe Freeman contributed to the design and facilitation of the café session, support of the student intern, oversight of data analysis, and manuscript preparation. Greig Taylor contributed to the design and facilitation of the café session, support of the student intern in focus groups, data collection and analysis, and manuscript preparation. Helen Rogers contributed to the design and facilitation of the café session and the manuscript preparation. Luisa Wakeling contributed the idea, design and facilitation of the café session, support and employment of the student intern, data analysis and manuscript preparation. All authors agreed the final manuscript.

## Funding

The authors have nothing to report.

## Ethics Statement

The study was approved by Newcastle University Research Ethics Committee (Ref: 24172/2022).

## Conflicts of Interest

The authors declare no conflicts of interest.

## Supporting information


**Supplementary Table 1.** The subthemes of ‘Knowledge’ and representative quotes from the four focus groups. Participants are indicated.

**Supplementary Table 2.** The subthemes of ‘Learning opportunities’ and representative quotes from the four focus groups. Participants are indicated.

**Supplementary Table 3.** The subthemes of ‘Delivery’ and representative quotes from the four focus groups. Participants are indicated.

**Supplementary Table 4.** The subthemes of ‘Improvements’ and representative quotes from the four focus groups. Participants are indicated.

## Data Availability

The data that support the findings of this study are available from the corresponding author upon reasonable request.
