# Supplementary material for: Caries Café: A Method for Consolidating and Co‐Constructing Knowledge of Caries
Source: Eur J Dent Educ. 2026 Mar 9;30(4):1482–91. doi: 10.1111/eje.70129 (PMC13686692; doi:10.1111/eje.70129)
Supplement: Supplementary file 1 [file EJE-30-1482-s001.docx]

| Learning opportunities within Caries Café | Beyond the curriculum | “… topics that you might not just consider just coming and seeing a normal patient or having a normal lecture…encouraged us to think a bit more outside the box …especially stations on sort of new technologies, like erm, kind of what could be done, like that doesn’t really get mentioned in lectures and stuff” | Year 3, Group 3, P4 |
| --- | --- | --- | --- |
|  | Future and wider implications | “The thing is, you go into something vocationally… you’re probably going to pay more attention to stuff that you know is going to be really helpful in the future.”  “… also, more into the future about research and stuff which I’d never really thought about before.”  “...the public health and government policy aspect of it, and future plans for public information and I thought that was pretty interesting and innovative.”  “Looking at sort of local and general population erm, issues, to do with dentistry so like accessing dental care or sort of issues keeping good oral hygiene, which I thought was interesting. I think it helps you when you sort of go and meet people from different backgrounds, different areas and thinking about the reason why you’re seeing in their mouth what you’re seeing.” | Year 5, Group 1, P2  Year 3, Group 3, P1  Year 3, Group 2, P1  Year 3, Group 2, P6 |
|  | Identifying caries | “I thought it was good because of the photos. Being able to see it properly cause we don’t, we’ve not had loads of experience looking at what it would look like erm, and obviously looks very different in different people and scenarios and everything so I think it was good to do that”  “…had loads of different pictures and you had to say, ‘was it caries?’...that was really helpful.” | Year 3, Group 2, P2  Year 3, Group 3, P3 |

**Supplementary Table 2.** The subthemes of ‘Learning opportunities’ and representative quotes from the four focus groups. Participants are indicated.
