# Supplementary material for: Caries Café: A Method for Consolidating and Co‐Constructing Knowledge of Caries
Source: Eur J Dent Educ. 2026 Mar 9;30(4):1482–91. doi: 10.1111/eje.70129 (PMC13686692; doi:10.1111/eje.70129)
Supplement: Supplementary file 2 [file EJE-30-1482-s002.docx]

| Theme | Context | Representative quote | Participant |
| --- | --- | --- | --- |
| Consolidation and co-construction of caries knowledge | Knowledge integration and confidence | “...some questions where I knew the answer but hadn’t thought about applying the knowledge in that way, so it was good to apply the knowledge I already had to lots of different areas and see how it all connects...”  “I think for me it was almost like a stepping-stone on to clinics. So obviously like we said it was all theory before and having cases to learn and kind of work from was good to perform obviously when we went to clinic.”  “Just like holistic subject knowledge and erm, topics related to caries as well. You feel more confident because you have that knowledge, you can answer the questions when patients ask.”  “For me the caries café was about tying together everything we’ve learned theoretically and applying it to clinical scenarios.” | Year 3, Group 2, P4  Year 5, Group 1, P5  Year 3, Group 2, P1  Year 5, Group 1, P1 |
|  | Consolidation of existing knowledge | “I feel like there wasn’t really anything there that we didn’t already know. Like it was just a refresher of knowledge...”  “I think if it was just in a lecture, I’d just kind of pass through that. But because we were all talking about it and like the reasons for that, that’s like consolidated in my mind now as if that was my real patient.”  “The caries café for me was, erm, putting things together and answering like more challenging questions that we would not think of.”  “I think in my head it helped in terms of tying things together and kind of encourage some more revision on the topic” | Year 3, Group 2, P3  Year 3, Group 3, P2  Year 5, Group 1, P4  Year 5, Group 1, P2 |
|  | Identification of gaps in knowledge | “…you find out oh I don’t actually know as much about it. So it can encourage you to revise a bit more on that subject…it was quite helpful to be given a kick like you should know this a bit better…you get the benefit of like the clinical stuff and then on the academic side you’re like oh flipping heck I don’t know anything about caries.”  “I mean I still think there’s, I need to like, do more to understand it better. I don’t feel like now I know everything there is to know but I think it was like a good start. And it reminds you of stuff you need to revise again” | Year 5, Group 1, P2  Year 3, Group 2, P2 |
|  | Co-construction of knowledge/ understanding | “It's one of those where you’re yet to see so many different opinions and have discussions. Like in lectures we’re told its useful for this, useful for that, whereas when you get to sit down and have a discussion or a debate about things, you realise that there are lots of different opinions that people use, like it's not just one thing set in stone, so I guess it kind of gives you scope to kind of explore different opinions and that's quite reassuring when you're in third year because you realise there's not actually a right or wrong way to do these things.”  “The caries café for me was really positive, I thought it was good. It was helpful like looking at caries from lots of different aspects, I thought it was good to see it from a range of different views really.”  “...more of a seminar type thing where you sit down, and you get the answer because the caries café is more everyone giving their opinion on things.”  “...it was to like blurt your ideas down from what you remember and get other people to fill in the gaps, I think that sort of helps as well with future memory of it with someone else, sort of, saying it is good little point in memory for future revision, like yeah, ‘so and so said that’.”  “It’s like a real-life scenario so it kind of, like it tests you because you’ve got to think about it, kind of, how you would if you were in that situation but then you can discuss like all the different options which is stuff you might not have thought of.”  “Yeah, some people will have done different reading to others so they can bring something new to the table.”  “Again, I don’t think we were really learning anything new particularly, I think it was just a nice sort of refresher, and just sort of to get other peoples’ experiences, like someone was saying that they had attrition and could recognise that in the child’s dental health image. It’s like they said that now I know when I see something like that, it’s cause they had it. It’s a nice way of picturing it all together.” | Year 5, Group 4 P3  Year 3, Group 2, P5  Year 5, Group 1, P2  Year 3, Group 2, P4  Year 3, Group 3, P1  Year 3, Group 3, P2  Year 3, Group 2, P4 |

**Supplementary Table 1.** The subthemes of ‘Knowledge’ and representative quotes from the four focus groups. Participants are indicated.
