# Supplementary material for: Caries Café: A Method for Consolidating and Co‐Constructing Knowledge of Caries
Source: Eur J Dent Educ. 2026 Mar 9;30(4):1482–91. doi: 10.1111/eje.70129 (PMC13686692; doi:10.1111/eje.70129)
Supplement: Supplementary file 3 [file EJE-30-1482-s003.docx]

| Delivery of Caries Café | Group work | “I think like working in groups was good cos everyone could sort of add to each other's thoughts. Yeah, it helps. Like if you’re stuck or something, someone else wants to add something and you’ll be like okay yeah that’s cool.”  “And the fact that we were like, not in the same group from start to finish like we were swapping over, it was like you can talk to people that you wouldn’t normally talk to generally, so it was quite nice in that aspect as well.”  “You know it's always it always ends up being like a few people who know the answers and then everyone else kind of sits back and feels a bit like don't know anything yeah. Maybe it was because we didn't have much knowledge or experience at the time.”  “ I mean it kind of it could two ways really couldn’t it, because you can have a bigger group and you'd feel less stressed out because that'd be more people to answer the question but, equally, that means you can often end up certain people can end up saying everything and some people sit back and not say anything, yeah smaller groups can be more intense as well can't make. And intensity isn't necessarily a bad thing” | Year 5, Group 1, P3  Year 3, Group 2, P3  Year 5, Group 4, P1  Year 5, Group 4, P3 |
| --- | --- | --- | --- |
|  | Learning dynamics | “...if there’s anything you have maybe a slight question about you can then ask that and then show that you have full knowledge of whatever you’re asking about erm, which obviously then helps you consolidate that if you have a full understanding of what the point is.”  “Really good. I think, as (P3) said it was really relaxed and everyone was really chatty so that just really helped, it was nice. [P3] Yeah, cos I feel like generally we are all quite chatty but when they ask questions in a lecture no one answers…”  “I think it’s always nice, well we haven't had in a long time, but when we're all a whole year group in one room and obviously we're working in groups and kind of moving around, I miss those days.”  “I think for me you get to work with people you wouldn’t normally work with”  “We got to see our friends. There were lots of people there that you don’t normally see on a day to day basis and like meeting lots of the lecturers that we haven’t met yet.”  “I think for me the caries cafe was, it was quite a long session. But it was quite good, but that was a lot of information in quite a short period of time.”  “I think the caries cafe was interesting but difficult at times and I found some of the tables a bit harder to work through” | Year 3, Group 2, P2  Year 3, Group 3, P2 and 3  Year 5, Group 4, P3  Year 5, Group 4, P1  Year 3, Group 2, P2  Year 5, Group 4, P2  Year 5, Group 4, P1 |
|  | The role of the facilitator | “The reason I remember it being different is because each of the stations was kind of from a different theoretical background so we had explanations from people in like the microbiology field, so I remember it being like a really rounded session.”  “Just the Interactions, and also like being able to ask questions. Cause they always say like, you can ask questions by emailing or say at the end, but no one really does, it’s a lot easier when you can just go there and ask a quick question.”  “You can say what you think and even if it's wrong, they can kind of explain why it’s not that, rather than why it’s a certain answer.”  “To just have like ‘have a good think, we're not going to judge, if you don't know just tell us’.  “I think even with like the lecturers, I guess when we normally interact with them it’s quite like a formal setting, so this was quite nice because it was quite informal.” | Year 5, Group 1, P1  Year 3, Group 2, P2  Year 5, Group 4, P4  Year 3, Group 3, P3  Year 3, Group 2, P3 |

**Supplementary Table 3.** The subthemes of ‘Delivery’ and representative quotes from the four focus groups. Participants are indicated.
