# Supplementary material for: Caries Café: A Method for Consolidating and Co‐Constructing Knowledge of Caries
Source: Eur J Dent Educ. 2026 Mar 9;30(4):1482–91. doi: 10.1111/eje.70129 (PMC13686692; doi:10.1111/eje.70129)
Supplement: Supplementary file 4 [file EJE-30-1482-s004.docx]

| Improvements to Caries Café | Student-generated notes | “Maybe like answers submitted into [learning platform] after the session so we know the ideal answers to the questions.” | Year 5, Group 1, P4 |
| --- | --- | --- | --- |
|  | Clinical relevance | “And if there was a table of a case study, [...] to sort of take us through the stages, that would, I think that would really, really help…especially in terms of investigations.”  “I think they could have had more stations to do with pictures of caries and discussing the actual process of it rather than…just because more experience of it would have definitely been more beneficial. Also, they could’ve had like..., actual teeth rather than photos, that would’ve been quite good”  “I think a lot of it was like, mind maps and just noting down your knowledge, but I think if it was more quiz based or something like that would’ve been pretty good as well, to see some diversity.”  “Maybe more ways to like to recognise it in like its early stages. Cause obviously it’s quite easy to recognise when there’s a massive hole in the tooth, but when it's just starting to become caries it's sometimes hard to know what that looks like or what to look for.” | Year 5, Group 4, P4  Year 3, Group 2, P2  Year 3, Group 2, P1  Year 3, Group 2, P4 |
|  | Other topics for café format | “Human Disease is pretty massive in its module so I think it would be pretty good to get everyone to, sort of, discuss it cause then you have people, your peers, explaining things to you as well at endo stations and that might be a lot easier to remember than just going to the lectures.”  “Maybe even like treatment planning in our conservation clinic” | Year 3, Group 2, P4  Year 3, Group 2, P1 |

**Supplementary Table 4.** The subthemes of ‘Improvements’ and representative quotes from the four focus groups. Participants are indicated.
